# Immunohistochemical Analysis of Single-Stranded DNA Binding Protein 2 in Non-Melanoma Skin Cancers

**DOI:** 10.3390/biomedicines11071818

**Published:** 2023-06-25

**Authors:** Seongsik Bang, Hwangkyu Son, Hyebin Cha, Kihyuk Song, Hosub Park, Hyunsung Kim, Joo Yeon Ko, Jaekyung Myung, Seungsam Paik

**Affiliations:** 1 Department of Pathology, Seoul Hospital, Hanyang University College of Medicine, Seoul 04763, Republic of Korea; grypony@naver.com (S.B.); ganzi4900@gmail.com (H.S.); chbin0111@gmail.com (H.C.); kihyuk1127@naver.com (K.S.); parkhstm@gmail.com (H.P.); hhnt5841@gmail.com (H.K.); 2 Department of Dermatology, Seoul Hospital, Hanyang University College of Medicine, Seoul 04763, Republic of Korea; drko0303@ehanyang.ac.kr

**Keywords:** non-melanoma skin cancer, basal cell carcinoma, squamous cell carcinoma, immunohistochemistry, single-stranded DNA binding protein 2, SSBP2

## Abstract

Single-stranded DNA binding protein 2 (SSBP2) is a tumor suppressor candidate. In this study, the expression level and clinicopathological significance of SSBP2 in squamous cell carcinoma (SCC) and basal cell carcinoma (BCC) were evaluated. We also identified biological pathways associated with a set of genes potentially related to SSBP2. Immunohistochemistry (IHC) was performed on 70 SCC and 146 BCC cases to assess SSBP2 expression semi-quantitatively. In addition, the associations between SSBP2 expression and clinicopathological characteristics were analyzed. Gene ontology (GO) enrichment analysis was performed using publicly available data and web-based bioinformatics tools. Compared with BCC, SCC had a significantly low SSBP2 expression (*p* < 0.001). In total, 12 (17.1%) of the 70 SCC cases and 30 (20.5%) of the 146 BCC cases showed low SSBP2 expression. Among SCC cases, ulceration (*p* = 0.005) and a deep level of invasion (*p* = 0.012) showed an association with low SSBP2 expression. Local recurrence was slightly more common in the SCC subgroup with low SSBP2 expression, although the difference was not significant (*p* = 0.058). Using GO enrichment analysis, we identified several biological functions performed by a set of 36 genes in SCC. SSBP2 evaluation using IHC can be helpful in the differential diagnosis of SCC and BCC. SSBP2 expression was associated with tumor invasiveness in SCC.

## 1. Introduction

Non-melanoma skin cancers (NMSCs) consist mostly of basal cell carcinoma (BCC) and squamous cell carcinoma (SCC), which are malignant neoplasms originating from epidermal keratinocytes [1]. NMSCs are treated by surgical removal, and patients generally have a good prognosis. However, the incidence of NMSCs is gradually increasing, and the consumption of medical resources for treatment is an essential health issue [2,3]. Although the pathogenesis of NMSCs is multifactorial, ultraviolet radiation from sunlight is a main etiological factor [4].

Single-stranded DNA binding protein 2 (SSBP2), also referred to as SSDP2, is a tumor suppressor candidate. Reduced expression of SSBP2 was detected in a human acute myelogenous leukemia (AML) cell line [5], and Liang et al. showed that SSBP2 might act as a regulator of cell proliferation and cell cycle [6]. SSBP2-null mice showed an enhanced predisposition to malignancy [7], and SSBP2 was downregulated in prostate cancer and esophageal squamous cell carcinoma cell lines and inhibited tumor cell growth [8,9]. Low SSBP2 expression at the protein level has been linked to an aggressive phenotype and unfavorable prognosis in some types of solid tumors [10,11,12,13]. However, studies on glioblastoma with an SSBP2-inherited variant [14] and hepatocellular carcinoma [15] showed that high SSBP2 expression was associated with poor clinical outcomes, suggesting an oncogenic role of SSBP2.

Immunohistochemistry (IHC) is a useful method for detecting a specific antigen using an antibody that can be visualized via staining and is used as an adjuvant for differential diagnosis or scientific research [16]. In clinical practice, IHC is performed for differential diagnosis of neoplastic skin diseases. Several diagnostic markers expressed in epidermal tumors (SCC and BCC) and sweat gland tumors have been identified [17]. There are many studies on biomarkers utilizing IHC to conduct risk stratification and to select appropriate treatments for SCC [18].

The precise role of SSBP2 as a tumor suppressor or promoter is unclear due to conflicting results across tumor types. The relationship between SSBP2 and NMSCs has rarely been studied. Inman et al. reported that SSBP2 was downregulated in SCC cell lines compared to normal human keratinocytes in gene expression analysis. In addition, the authors performed a bioinformatic analysis using public data sets in actinic keratosis and SCC. They identified that SSBP2 was downregulated at the mRNA level, indicating that SSBP2 may be involved in tumor suppression [19]. However, the expression of SSBP2 in BCC, which accounts for the largest number of NMSC cases, has not been reported.

Therefore, we intended to identify SSBP2 expression in SCC and BCC using IHC and to determine whether differences in SSBP2 expression could help in the differential diagnosis of tumors. In addition, the clinicopathological significance was investigated to reveal the functional role of SSBP2. We identified biological pathways involving a set of genes potentially related to SSBP2 using publicly available data and web-based bioinformatics tools.

## 2. Materials and Methods

### 2.1. Study Design and Clinical Data Collection

We included patients diagnosed with SCC or BCC at Hanyang University Hospital (Seoul, Republic of Korea) between September 2008 and June 2019. According to the Declaration of Helsinki principles, approval was obtained from the Institutional Review Board of Hanyang University Hospital before conducting the study (HYU 2019-10-062, approval date: 15 January 2020). Most of the specimens used in this study were collected before February 2013, and a database able to distinguish patients by case number only without viewing personally identifiable information was constructed. The requirement for informed consent was waived because this study involved minimal risk to participants and did not infringe on the participants’ rights. Specimens collected after February 2013 were from subjects who participated voluntarily. Totals of 75 SCC and 174 BCC cases were retrospectively enrolled; 5 SCC and 28 BCC cases without sufficient tumor tissue were excluded. The following clinical information used in the analysis was obtained from a review of electronic medical records for age, sex, tumor location, ulceration, local recurrence, and lymph node metastasis.

### 2.2. Pathological Evaluation

All tissue slides used at diagnosis were reviewed to determine pathological characteristics, including histological subtypes of BCC, histological grade of SCC, level of invasion, and perineural invasion. Micronodular, infiltrating, sclerosing/morphoeic, and basosquamous patterns were classified as histopathologically aggressive BCC subtypes [20,21]. The histological grade of SCC was divided using a three-level grading system according to several morphologic features of differentiation (nuclear pleomorphism, degree of keratinization) [22]. The presence of ulceration was determined by clinical data rather than microscopic findings in order not to include the biopsy site.

### 2.3. SSBP2 Immunohistochemistry

We first constructed tissue microarrays (TMAs) to detect the molecular target in many specimens at once [23]. A representative portion of the tumor in each case was selected during the tissue slide review. The cylindrical cores (measuring 3.0 mm in diameter) were acquired from formalin-fixed, paraffin-embedded tissue blocks (donor blocks) corresponding to the tissue slides and were deposited on recipient TMA blocks (Unitma, Gyeonggi-do, Republic of Korea). Each TMA block consisted of 6 × 5 tumor samples.

TMA blocks were cut into 4 μm thick sections and underwent IHC using the Benchmark XT automated staining system (Ventana Medical Systems, Tucson, AZ, USA) according to the manufacturer’s protocol. Heat-induced epitope retrieval was performed with CC1 Tris-EDTA buffer (Ventana Medical Systems, Tucson, AZ, USA). The OptiView DAB IHC Detection Kit (Ventana Medical Systems, Tucson, AZ, USA) was used to block endogenous peroxidase and detect antigen–antibody complexes. Counterstaining was performed using modified Mayer’s hematoxylin (Hematoxylin II). Rabbit recombinant monoclonal SSBP2 antibody (diluted 1:200) (ab177944; Abcam, Cambridge, UK) was used as the primary antibody.

### 2.4. Assessment of SSBP2 Expression

**Figure 1:**
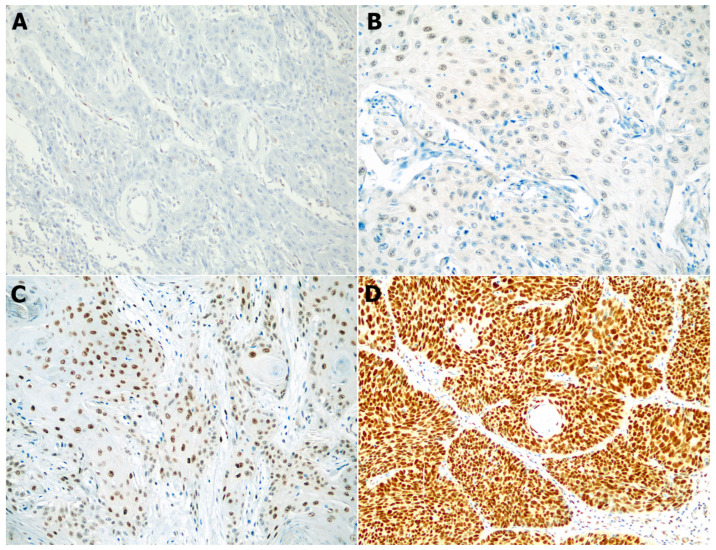
Representative images of SSBP2 expression in squamous cell carcinoma (200×). Negative (**A**), weak (**B**), moderate (**C**), and strong (**D**) nuclear expression.

SSBP2 expression was evaluated independently by two pathologists (S.B. and S.P.), and nuclear staining of tumor cells was considered indicative of positive SSBP2 expression. We calculated the histoscore (H-score) based on staining intensity and the percentage of stained tumor cells. The intensity of IHC staining was classified as negative (0), weak (1+), moderate (2+), or strong (3+). Representative microphotographs of SSBP2 staining are shown in Figure 1. In each case, the H-score was calculated as follows: H-score = [(1 × % weakly stained) + (2 × % moderately stained) + (3 × % strongly stained)]. There is no standard cutoff for SSBP2 expression of NMSC. Therefore, we performed receiver operating characteristic (ROC) curve analysis and determined the optimal cutoff value as the point maximizing Youden’s index in each of the BCC and SCC groups.

### 2.5. Statistical Analyses

We used SPSS version 25.0 (IBM Corporation, Armonk, NY, USA) for statistical analyses. The H-score of NMSC cases is a continuous variable with a non-normal distribution, and the Mann–Whitney U test was used to evaluate the mean difference in SSBP2 expression between the SCC and BCC tissue groups. NMSC cases were classified into high and low SSBP2 expression groups, and each clinicopathological characteristic was divided into two categories. Pearson’s Chi-square (χ2) or Fisher’s exact test was performed in two-by-two contingency tables to reveal the associations between SSBP2 expression and clinicopathological characteristics.

### 2.6. Functional Analysis

We performed gene ontology (GO) enrichment analysis to identify differentially expressed genes (DEGs) between the low and high SSBP2-expression groups and to reveal the related biological functions. To obtain gene expression data of SCC, GSE45216 (https://www.ncbi.nlm.nih.gov/geo/; accessed on 17 April 2023) deposited in the gene expression omnibus (GEO) database was utilized. GSE45216 includes RNA expression data collected using microarray analysis of 30 samples of SCC. We used the GEO2R tool to select the top 250 candidates showing significant expression differences between the low and high SSBP2-expression groups (https://www.ncbi.nlm.nih.gov/geo/geo2r/; accessed on 18 April 2023). Several genes were detected by multiple probes, and a particular one among them showing stronger concordance was used for this study [24]. Then, 39 genes that satisfied the cutoff value (log2FoldChange ≥ 1 or log2FoldChange ≤ −1, *p* value < 0.05) were identified as DEGs. We performed GO enrichment analysis using the Database for Annotation, Visualization, and Integrated Discovery (DAVID) (https://david.ncifcrf.gov/home.jsp; accessed on 18 April 2023) [25]. In functional annotation, *p* value < 0.05 was considered statistically significant.

## 3. Results

### 3.1. Clinicopathological Characteristics

The median age of SCC patients was 77 years (range, 33–96 years), while that of BCC patients was 71 years (range, 29–98 years). The SCC group included 34 women and 36 men, while the BCC group included 86 women and 60 men. Most SCCs were located on the face (54.3%), upper/lower extremities (25.7%), and scalp (10%), while the majority of BCCs were located on the face (79.4%), trunk (7.5%), and scalp (4.8%). Ulceration was identified in 21 SCC cases (30.0%) and 18 BCC cases (12.3%). Nodular and mixed subtypes were the main histological subtypes of BCC (60.2% and 20.5%, respectively), and most SCCs were well-differentiated (74.3%). The majority of SCC and BCC cases showed invasion only above the subcutaneous tissue (71.4% and 77.4%, respectively). The clinicopathological characteristics of SCC and BCC are summarized in Table S1.

### 3.2. Differences in SSBP2 Expression between SCC and BCC

**Figure 2:**
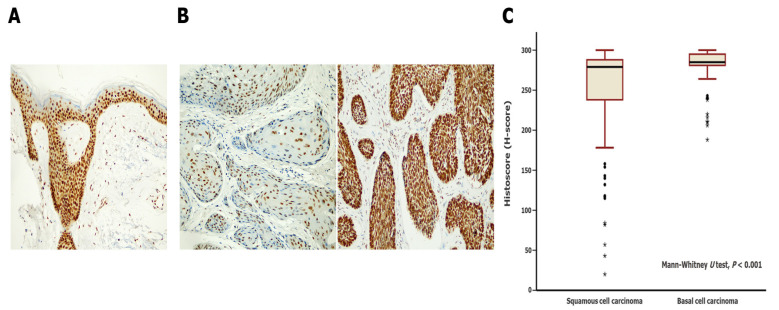
Comparison of SSBP2 expression in squamous cell carcinoma (SCC) and basal cell carcinoma (BCC). (**A**) Normal surface and follicular epitheliums showed diffuse and strong SSBP2-positive staining (200×). (**B**) BCC samples showed more diffuse and stronger positive staining than SCC samples (200×). (**C**) According to the Mann–Whitney U test, the mean H-score rank of SCC cases was statistically lower than that of BCC cases (*p* < 0.001). Circles and stars represent outliers.

We evaluated SSBP2 expression in 70 SCC and 146 BCC cases. H-scores are presented as mean ± standard deviation. The overall mean H-score of NMSCs was 270.6 (±48.2) points, while the mean H-score of SSBP2 expression was 245.4 (±73.6) points in the SCC group and 282.6 (±20.4) points in the BCC group. The mean rank of the H-score among SCC cases was significantly lower than that among BCC cases (*p* < 0.001, Mann–Whitney U test). Differences in SSBP2 expression between SCC and BCC cases are presented in Figure 2.

### 3.3. Clinicopathological Implications of Low SSBP2 Expression in SCC and BCC

SCC cases were categorized into high and low SSBP2-expression groups using ROC curve analysis (H-score > 155 points vs. H-score ≤ 155 points). Accordingly, 12 cases (17.1%) were included in the low SSBP2-expression group. Low SSBP2 expression was significantly associated with ulceration (*p* = 0.005) and a deep level of invasion (*p* = 0.012) among SCC cases. Local recurrence was also more frequent in the low SSBP2-expression group; however, this trend was not statistically significant (*p* = 0.058). No significant influence of differences in SSBP expression was found in age, sex, tumor size, sun exposure, perineural invasion, lymph node metastasis, and histological grade. We present the association between SSBP2 expression of SCC cases and clinicopathological characteristics in Table 1. BCC cases were categorized into high and low SSBP2-expression groups using ROC curve analysis (H-score > 275 points vs. H-score ≤ 275 points). Thirty cases (20.5%) were included in the low SSBP2-expression group. However, the expression level of SSBP2 did not show a significant association with the clinicopathological characteristics of BCC cases. We present the association between SSBP2 expression of BCC cases and clinicopathological characteristics in Table 2.

### 3.4. Enrichment Analysis on SSBP2-Related Gene Sets in SCC

**Figure 3:**
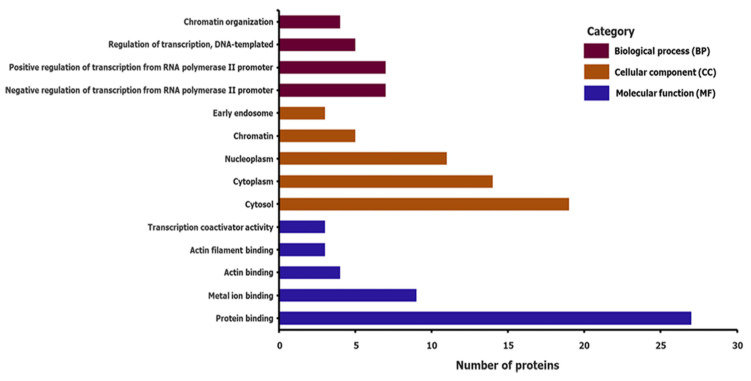
Biological pathways identified using gene ontology enrichment analyses.

GO enrichment analysis was performed for the gene set using DAVID 6.8, a functional annotation tool. We considered 39 genes as targets, and 3 genes not found in the DAVID database were excluded from the analysis. GO consists of the following three biological domains: molecular function (MF), cellular component (CC), and biological process (BP). For the MF category, five GO terms were associated with the target genes: protein binding (GO:0005515), metal ion binding (GO:0046872), actin binding (GO:0003779), actin filament binding (GO:0051015), and transcription coactivator activity (GO:0003713). The CC category revealed by GO enrichment analysis includes cytosol (GO:0005829), cytoplasm (GO:0005737), nucleoplasm (GO:0005654), chromatin (GO:0000785), and early endosome (GO:0005769). The BP category was dominated by the negative regulation of transcription from RNA polymerase II promoter (GO:0000122), the positive regulation of transcription from RNA polymerase II promoter (GO:0045944), the regulation of DNA-templated transcription (GO:0006355), and chromatin organization (GO:0006325). The results of the GO enrichment analyses are presented in Figure 3. Genes involved in biological functions are summarized in Table S2.

## 4. Discussion

This study was designed to evaluate SSBP2 expression using IHC in NMSCs and to investigate its clinicopathological significance. We found a significant difference in SSBP2 expression levels between the SCC and BCC groups (*p* < 0.001). In addition, we identified low SSBP2 expression to be significantly associated with ulceration (*p* = 0.005) and a deep level of invasion (*p* = 0.012) in SCC. Using bioinformatics tools, we revealed several biological functions involving a set of genes potentially related to SSBP2 in SCC.

Many cases of SCC typically develop from precursor lesions on sun-exposed skin [26]. AK and squamous cell carcinoma in situ are representative precursor lesions of SCC and exhibit significant histopathologic similarities to SCC [27]. In addition, recent studies have revealed genomic similarities, such as NOTCH1 and TP53, between these tumors [28,29]. Although the high tumor mutation burden of SCC makes it challenging to identify key driver genes, several clinical trials are being conducted for various molecular targets [30]. The leading cause of BCC is UV exposure, and several genetic syndromes are related to inherited susceptibility [31,32]. The dysregulation of the hedgehog signaling pathway is a major molecular mechanism of BCC [33]. The therapies targeting this downstream signaling cascade are under investigation [34,35].

Several studies investigating the association of SSBP2 with development have been reported. Meyel et al. reported that knockout mice for SSBP2 showed embryonic lethality [36]. Xu et al. [37] and Li et al. [38] revealed that SSBP2 is involved in the differentiation of erythroid progenitors and in hematopoietic and progenitor stem cell homeostasis, respectively. During development, SSBP2 positively regulates LIM domain binding 1 (LDB1) and LIM-domain-only 2 (LMO2) proteins, inhibiting degradation [37,39]. Altered expression of both LDB1 and LMO2 has been documented in various tumor types [40,41]. In addition, as oncogenic roles of LDB1 and LMO2 have been suggested [42,43], SSBP2 could contribute to tumorigenesis.

The mechanisms regulating SSBP2 expression in tumor cells remain unclear. Castro et al. reported that SSBP2, located at 5q, is the apparent target of unbalanced translocations and deletions in leukemia, but no inactivating mutation was found [5]. Many studies have identified the promoter hypermethylation of SSBP2 in several types of solid cancers, suggesting that epigenetic alteration may be related to the downregulation of SSBP2. Liu et al. revealed the promoter hypermethylation of SSBP2 in prostate cancer (61%) and an association between hypermethylation status and tumor stage [8]. Huang et al. reported promoter hypermethylation (86%) and downregulation of SSBP2 in esophageal cancer [9]. Kagohara et al. reported promoter hypermethylation in GBC (52.6%) compared to cholecystitis (0%) [44], and Brait et al. identified promoter hypermethylation in 9% of ovarian cancer cases [45].

This study has several limitations. First, we conducted a retrospective study at a single institution and included a limited number of NMSC cases. Second, survival analysis according to the expression level of SSBP2 could not be performed because deaths closely related to NMSCs rarely occur. Thirdly, SSBP2 expression in NMSCs was investigated using only the IHC method. We evaluated SSBP2 expression at the protein level and confirmed that SSBP2 expression in SCC was related to tumor invasiveness. However, in vitro/in vivo experiments are needed to establish the functional roles of SSBP2 in cell invasion, migration, and proliferation. In addition, the biological pathways involved in SSBP2 expression cannot be clearly explained by the IHC method; instead, we were able to identify a biological pathway in a set of genes potentially related to SSBP2 using bioinformatics analysis, and further experimental studies are needed to confirm the exact molecular pathways associated with SSBP2.

In conclusion, there was a significant difference in SSBP2 expression between SCC and BCC, suggesting its potential as a tool for differential diagnosis. Additionally, we revealed the association of SSBP2 expression with tumor invasiveness in SCC.

## Figures and Tables

**Table 1:** Clinicopathological characteristics according to the expression level of SSBP2 in patients with squamous cell carcinoma (*n* = 70).

Parameters	SSBP2 Expression		*p* Value
	High Expression (%) (*n* = 58)	Low Expression (%) (*n* = 12)	
Age			0.490 *
<70 years	14 (77.8%)	4 (22.2%)	
≥70 years	44 (84.6%)	8 (15.4%)	
Sex			0.112 *
Female	31 (91.2%)	3 (8.8%)	
Male	27 (75.0%)	9 (25.0%)	
Tumor size ^†^			0.148
<2.0 cm	37 (84.1%)	7 (15.9%)	
≥2.0 cm	10 (66.7%)	5 (33.3%)	
Location			0.678 *
Sun-protected	8 (80%)	2 (20%)	
Sun-damaged	50 (83.3%)	10 (16.7%)	
Ulceration			0.005 *
Not identified	45 (91.8%)	4 (8.2%)	
Present	13 (61.9%)	8 (38.1%)	
Perineural invasion			1.000 *
Not identified	54 (81.8%)	12 (18.2%)	
Present	4 (100%)	0 (0%)	
Local recurrence			0.058 *
No	55 (85.9%)	9 (14.1%)	
Yes	3 (50.0%)	3 (50.0%)	
Lymph node metastasis			0.133 *
No	56 (84.8%)	10 (15.2%)	
Yes	2 (50.0%)	2 (50.0%)	
Histological grade			0.166 *
Grade 1	41 (78.8%)	11 (21.2%)	
Grade 2 or 3	17 (94.4%)	1 (5.6%)	
Level of invasion			0.012
Above subcutis	45 (90.0%)	5 (10.0%)	
Below subcutis	13 (65.0%)	7 (35.0%)	

* Fisher’s exact test. ^†^ Tumor size; 11 cases were missing data.

**Table 2:** Clinicopathological characteristics according to the expression level of SSBP2 in patients with basal cell carcinoma (*n* = 146).

Parameters	SSBP2 Expression		*p* Value
	High Expression (%) (*n* = 116)	Low Expression (%) (*n* = 30)	
Age			0.498
<70 years	50 (76.9%)	15 (23.1%)	
≥70 years	66 (81.5%)	15 (18.5%)	
Sex			0.266
Female	71 (82.6%)	15 (17.4%)	
Male	45 (75.0%)	15 (25.0%)	
Tumor size ^†^			1.000 *
<2.0 cm	81 (80.2%)	20 (19.8%)	
≥2.0 cm	10 (83.3%)	2 (16.7%)	
Location			0.765 *
Sun-protected	16 (84.2%)	3 (15.8%)	
Sun-damaged	100 (78.7%)	27 (21.3%)	
Ulceration			1.000 *
Not identified	101 (78.9%)	27 (21.1%)	
Present	15 (83.3%)	3 (16.7%)	
Perineural invasion			0.187 *
Not identified	114 (80.3%)	28 (19.7%)	
Present	2 (50.0%)	2 (50.0%)	
Local recurrence			0.581 *
No	112 (78.9%)	30 (21.1%)	
Yes	4 (100%)	0 (0%)	
Histological subtypes ^‡^			0.413
Less aggressive and others	86 (81.1%)	20 (18.9%)	
Aggressive	30 (75.0%)	10 (25.0%)	
Level of invasion			0.224 *
Above subcutis	87 (77.0%)	26 (23.0%)	
Below subcutis	29 (87.9%)	4 (12.1%)	

* Fisher’s exact test. ^†^ Tumor size; 33 cases were missing data. ^‡^ Less aggressive and others: nodular, superficial, fibroepithelial, and BCC with adnexal differentiation. Aggressive: micronodular, infiltrating, sclerosing/morphoeic, and basosquamous. Abbreviation: SSBP2, single-stranded DNA binding protein 2.

## Data Availability

Publicly available datasets were analyzed in this study. These data can be found here: [https://www.ncbi.nlm.nih.gov/geo/query/acc.cgi?acc=%20GSE45216; accessed on 17 April 2023].

## Supplementary Materials

The following supporting information can be downloaded at: https://www.mdpi.com/article/10.3390/biomedicines11071818/s1, Table S1: Clinicopathological characteristics of patients with squamous cell carcinoma and basal cell carcinoma; Table S2: Biological functions involving a set of genes potentially related to SSBP2 in squamous cell carcinoma derived by gene ontology enrichment analysis.
