# Supplementary material for: Immunohistochemical Analysis of Single-Stranded DNA Binding Protein 2 in Non-Melanoma Skin Cancers
Source: Biomedicines. 2023 Jun 25;11(7):1818. doi: 10.3390/biomedicines11071818 (PMC10376428; doi:10.3390/biomedicines11071818)
Supplement: Supplementary file 1 [file biomedicines-11-01818-s001.zip › biomedicines-2426816-supplementary.pdf]

**Supplementary Table S1.** Clinicopathological characteristics of patients with squamous cell carcinoma and basal cell carcinoma

| Characteristics | SCC (n = 70) | BCC (n = 146) |
|---|---|---|
| Age, median (range, years) | 77 (33–96) | 71 (29–98) |
| Sex | | |
| Female | 34 (48.6%) | 86 (58.9%) |
| Male | 36 (51.4%) | 60 (41.1%) |
| Tumor size, mean (range, cm)* | 2.3 (0.2–14.0) | 1.1 (0.2–5.0) |
| Location | | |
| Scalp | 7 (10.0%) | 7 (4.8%) |
| Face | 38 (54.3%) | 116 (79.4%) |
| Neck | 1 (1.4%) | 2 (1.4%) |
| Upper extremity | 7 (10.0%) | 1 (0.7%) |
| Lower extremity | 11 (15.7%) | 3 (2.1%) |
| Trunk | 6 (8.6%) | 11 (7.5%) |
| Urogenital region | 0 (0%) | 6 (4.1%) |
| Ulceration | | |
| Not identified | 49 (70.0%) | 128 (87.7%) |
| Present | 21 (30.0%) | 18 (12.3%) |
| Perineural invasion | | |
| Not identified | 66 (94.3%) | 142 (97.3%) |
| Present | 4 (5.7%) | 4 (2.7%) |
| Local recurrence | | |
| No | 64 (91.4%) | 142 (97.3%) |
| Yes | 6 (8.6%) | 4 (2.7%) |
| Lymph node metastasis | | |
| No | 66 (94.3%) | 146 (100%) |
| Yes | 4 (5.7%) | 0 (0%) |
| Histological subtype | | |
| Nodular | | 88 (60.2%) |
| Superficial | | 11 (7.5%) |
| Micronodular | | 8 (5.5%) |
| Infiltrating | | 2 (1.4%) |
| Sclerosing/morphoeic | | 1 (0.7%) |
| Basosquamous | | 2 (1.4%) |
| Fibroepithelial | | 1 (0.7%) |
| BCC with adnexal differentiation | | 3 (2.1%) |
| Mixed | | 30 (20.5%) |

| | | |
|---|---|---|
| Histological grade | | |
| G1 (well differentiated) | 52 (74.3%) | |
| G2 (moderately differentiated) | 15 (21.4%) | |
| G3 (poorly differentiated) | 3 (4.3%) | |
| Level of invasion | | |
| Papillary dermis | 16 (22.8%) | 12 (8.2%) |
| Reticular dermis | 34 (48.6%) | 101 (69.2%) |
| Subcutis | 14 (20.0%) | 20 (13.7%) |
| Skeletal muscle | 6 (8.6%) | 13 (8.9%) |

* Tumor size; 11 cases of SCC and 33 cases of BCC were missing data.

Abbreviations: BCC, basal cell carcinoma; SCC, squamous cell carcinoma.

**Supplementary Table S2.** Biological functions involving a set of genes potentially related to SSBP2 in squamous cell carcinoma derived by Gene Ontology enrichment analysis

| GO category | Term | Genes | Fold enrichment |
|---|---|---|---|
| MF | Actin filament binding (GO:0051015) | *MYO10*, *MYO9A*, *MYOM3* | 7.48 |
| | Actin binding (GO:0003779) | *MYO10*, *KLHL3*, *PDLIM5*, *MYO9A* | 6.51 |
| | Transcription coactivator activity (GO:0003713) | *NCOA1*, *KAT6B*, *SUB1* | 6.30 |
| | Metal ion binding (GO:0046872) | *ZFYVE16*, *KAT6B*, *JMJD1C*, *WDFY2*, *PDLIM5*, *JAK2*, *MYO9A*, *FOXP2*, *BPTF* | 1.87 |
| | Protein binding (GO:0005515) | *OTCH3*, *GALT*, *UBA6*, *JMJD1C*, *PTPN22*, *NR3C1*, *FAM124B*, *APBB2*, *JAK2*, *FNIP1*, *PDLIM5*, *BPTF*, *NCOA1*, *CAST*, *MYO10*, *KLHL3*, *MYO9A*, *KRT74*, *FOXP2*, *SHISA6*, *ZFYVE16*, *KAT6B*, *SUB1*, *STRAP*, *SNRPF*, *WDFY2*, *FAM50A* | 1.19 |
| CC | Early endosome (GO:0005769) | *ZFYVE16*, *APBB2*, *WDFY2* | 5.86 |
| | Chromatin (GO:0000785) | *NCOA1*, *JMJD1C*, *NR3C1*, *FOXP2*, *BPTF* | 2.87 |
| | Cytosol (GO:0005829) | *NCOA1*, *CAST*, *NOTCH3*, *GALT*, *MYO10*, *UBA6*, *HSD17B4*, *KLHL3*, *PTPN22*, *NR3C1*, *MYO9A*, *KRT74*, *ZFYVE16*, *CDC42EP5*, *STRAP*, *SNRPF*, *JAK2*, *FNIP1*, *PDLIM5* | 2.08 |
| | Nucleoplasm (GO:0005654) | *NCOA1*, *NOTCH3*, *FAM124B*, *KAT6B*, *SUB1*, *JMJD1C*, *SNRPF*, *JAK2*, *NR3C1*, *BPTF*, *FAM50A* | 1.68 |
| | Cytoplasm (GO:0005737) | *CAST*, *GALT*, *UBA6*, *PTPN22*, *NR3C1*, *MYO9A*, *KRT74*, *CDC42EP5*, *STRAP*, *APBB2*, *JAK2*, *FNIP1*, *PDLIM5*, *BPTF* | 1.50 |
| BP | Chromatin organization (GO:0006325) | *JMJD1C*, *JAK2*, *NR3C1*, *FAM50A* | 8.22 |
| | Negative regulation of transcription from RNA polymerase II promoter | *NOTCH3*, *STRAP*, *APBB2*, *FNIP1*, *NR3C1*, *FOXP2*, *BPTF* | 4.05 |

| | | | |
|---|---|---|---|
| | (GO:0000122) | | |
| | Positive regulation of transcription from RNA polymerase II promoter (GO:0045944) | *NCOA1*, *NOTCH3*, *KAT6B*, *APBB2*, *JAK2*, *NR3C1*, *BPTF* | 3.31 |
| | Regulation of transcription, DNA-templated (GO:0006355) | *KAT6B*, *JMJD1C*, *APBB2*, *NR3C1*, *BPTF* | 2.92 |

Abbreviations: BP, biological process; CC, cellular component; MF, molecular function; SSBP2, single-stranded DNA binding protein 2.
